# The traditional Chinese medicine manipulation combined with Schroth exercises for adolescent idiopathic scoliosis: protocol for a randomized controlled trial

**DOI:** 10.3389/fped.2025.1693246

**Published:** 2025-12-04

**Authors:** Jun Tao, Hongjian Li, Guilong Zhang, Rong Hu, Tangyu Liao, Fei Wang, Zi Yin, Wenqi Feng

**Affiliations:** 1 Rehabilitation Department, Yibin Traditional Chinese Medicine Hospital, Yibin, China; 2 Orthopedics Department, Hospital of Chengdu University of Traditional Chinese Medicine, Chengdu, China

**Keywords:** scoliosis, exercise therapy, traditional Chinese medicine, manipulation, cobb angle, thermography, randomized controlled trials

## Abstract

**Introduction:**

Adolescent idiopathic scoliosis (AIS) is a three-dimensional spinal deformity that can impair posture, function, and quality of life. Schroth exercise, a physiotherapeutic scoliosis-specific exercise, has demonstrated benefits in reducing Cobb angle and improving patient-reported outcomes. Traditional Chinese Medicine (TCM) manipulation may modulate musculoskeletal asymmetry and pain, potentially exerting synergistic effects when combined with Schroth therapy. This study aims to evaluate whether TCM manipulation plus Schroth exercise is superior to Schroth exercise alone in improving spinal curvature, patient-centered outcomes, and paraspinal symmetry in AIS.

**Methods:**

This is a single-center, randomized controlled, two-arm parallel-group superiority trial. Sixty adolescents (aged 10–18) with idiopathic scoliosis and Cobb angle 10°–45° will be randomized 1:1 to: (1) Schroth therapy alone (control) or (2) TCM manipulation combined with Schroth exercise (intervention). Primary outcome is change in Cobb angle measured by full-length standing anteroposterior spinal radiograph before and after the intervention. Secondary outcomes include Angle of Trunk Rotation (ATR), paraspinal thermal asymmetry assessed by infrared thermography, and health-related quality of life measured by the Scoliosis Research Society-22r (SRS-22r) questionnaire. Participants will be randomly allocated (1:1) using a computer-generated, stratified block randomization sequence with allocation concealed by sealed opaque envelopes. The analysis will follow the intention-to-treat (ITT) principle, and an ANCOVA adjusting for baseline measurements will be used for between-group comparisons. A total sample size of 60 participants (30 per group) was determined *a priori* to provide 80% power (*α* = 0.05, *β* = 0.20) to detect a clinically meaningful difference in Cobb angle, allowing for a 20% attrition rate.

**Discussion:**

If effective, the combined regimen could offer a non-invasive, integrative therapeutic option for AIS, with implications for conservative management guidelines.

**Ethics and dissemination:**

The study protocol was approved by the Ethics Committee of Yibin Traditional Chinese Medicine Hospital [Approval No. KY2024 Review (016)]. The trial has been prospectively registered in the International Traditional Medicine Clinical Trial Registry (ITMCTR) on January 6, 2025. The study will be conducted in accordance with the principles of the Declaration of Helsinki (Edinburgh 2000 revision). The trial findings will be disseminated through publication in peer-reviewed journals.

**Trial registration:**

The full trial protocol and statistical analysis plan can be accessed via the ITMCTR website (Registration No. ITMCTR2025000131). Any important modifications to the protocol will be submitted for approval by the institutional ethics committee and updated in the ITMCTR.

## Sponsor

Yibin Traditional Chinese Medicine Hospital (xu13909092923@163.com).

## 1 Introduction

Adolescent idiopathic scoliosis (AIS) is a structural, lateral, three-dimensional spinal deformity with onset during growth spurts in adolescence (1). If left untreated, moderate curves can progress, leading to cosmetic concerns, back pain, and psychosocial impairment (2). Severe untreated curves may progress and result in cardiorespiratory compromise (3). Conservative management, especially for mild-to-moderate curves, aims to halt progression and improve function without surgery (4). Schroth therapy is a scoliosis-specific exercise program focusing on three-dimensional corrective postural training, breathing techniques, and sensorimotor re-education. Evidence from randomized controlled studies indicates that Schroth exercises can reduce Cobb angle and improve trunk symmetry and quality of life in AIS patients (5–7).

Complementary therapies, including manual modalities, have been explored to enhance conservative management. Recent integration of manual therapies with scoliosis-specific exercise has shown promise; for example, combinations of spinal manipulation with Schroth have been evaluated with preliminary favorable findings (8, 9). Similarly, in China, Traditional Chinese Medicine (TCM) manipulation therapies such as Tuina are commonly used as conservative interventions for scoliosis (10). This approach employs soft-tissue relaxation techniques and spinal joint adjustments to rebalance paraspinal musculature and improve spinal alignment (11). This may alleviate pain and contribute to better overall patient outcomes.

However, current evidence remains limited. Most existing studies investigating manual therapies for scoliosis are preliminary, with small sample sizes, heterogeneous interventions, and methodological shortcomings that restrict the generalizability of their findings (12). Therefore, this study is designed to test the hypothesis that combining TCM manipulation with the Schroth method will yield superior improvements in spinal curvature, postural control, and pain reduction compared with the Schroth therapy alone. This trial aims to provide rigorous clinical evidence for the integration of TCM manipulation into evidence-based conservative management of AIS.

## 2 Materials and methods

### 2.1 Study design

This is a single-center, two-arm, parallel-group, randomized controlled trial. The study is being conducted at Yibin Traditional Chinese Medicine Hospital in Sichuan Province, China. This hospital is a designated National Center for Scoliosis, ensuring an adequate pool of eligible participants to achieve the target sample size within the planned timeframe. The overall trial recruitment period spans from June 1, 2025, to December 31, 2025, following ethical approval (granted in 2024) and prospective trial registration (January 6, 2025). To facilitate recruitment, advertisements have been placed in prominent locations within the outpatient hall of Yibin Hospital of Traditional Chinese Medicine and disseminated through the hospital's official WeChat account. There was no direct involvement of patients or the public in the design, conduct, or reporting of this trial. Public involvement was limited to dissemination of recruitment advertisements through the hospital's official WeChat account and its redistribution by community members.

The sponsor, Yibin TCM Hospital, is responsible for study initiation and oversight. The sponsor provided input on study design and facilitates trial management. However, all data collection is being carried out, and all future data analysis and interpretation will be performed, by the research team independently. The funder (Yibin Science and Technology Department) had no role in the study design and will have no role in the conduct, analysis, or decision to publish this study.

**Figure 1:**
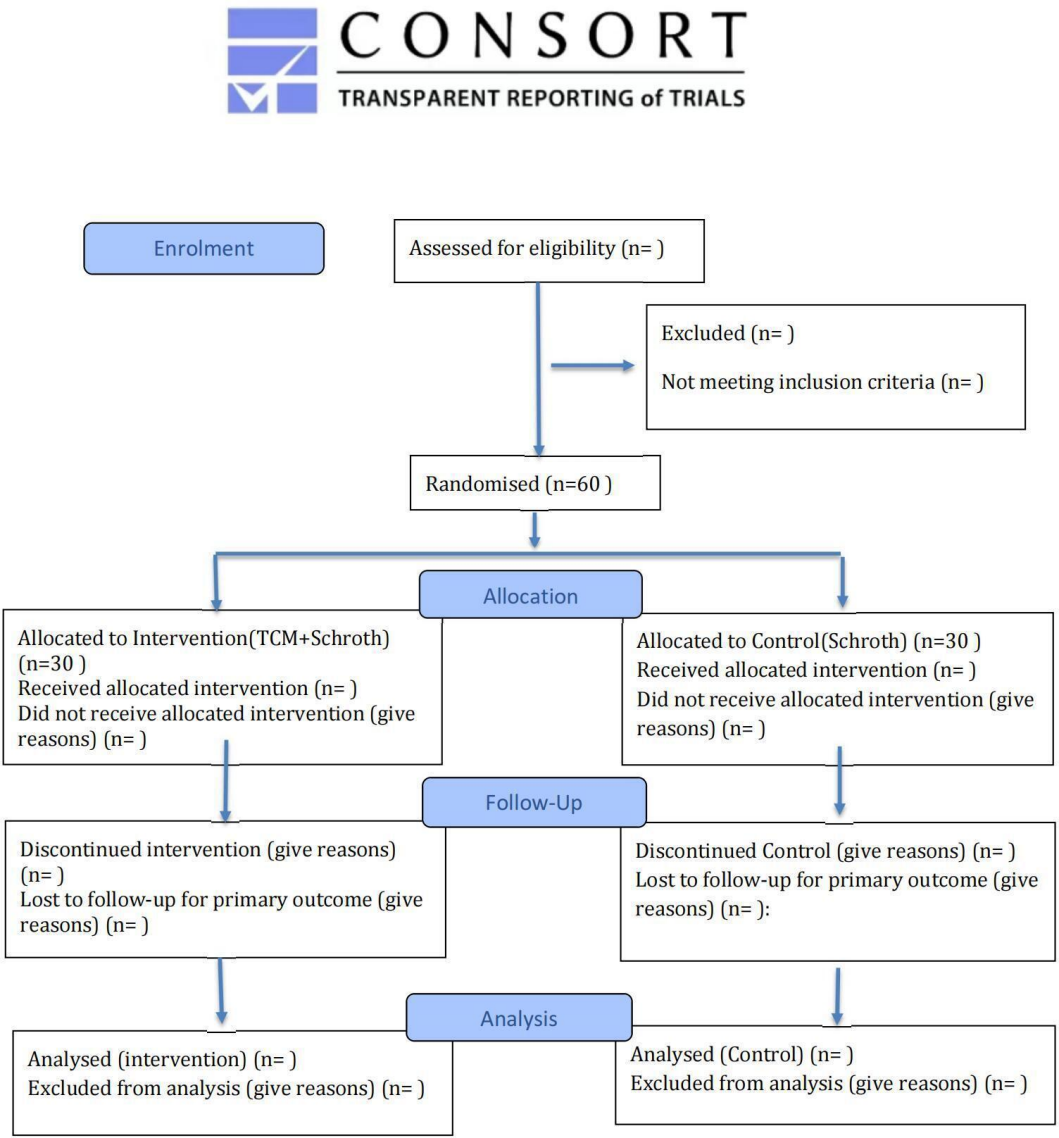
CONSORT flow diagram.

The study protocol was developed in accordance with the SPIRIT 2025 statement (13) and will be reported following the CONSORT (Consolidated Standards of Reporting Trials) guidelines. A completed SPIRIT checklist is provided as Supplementary Material. For each individual participant, the study timeline is defined relative to the date of randomization (designated as Week 0). The schedule of enrollment, interventions, and assessments for each participant is detailed in Table 1, and participant flow will be documented using a CONSORT diagram (Figure 1).

**Table 1:** Schedule of enrollment, interventions, and assessments (SPIRIT schedule).

		STUDY PERIOD				
		Enrollment	Allocation	Post allocation	Close-out	Follow-up
TIMEPOINT	Week	0	0	1	2	12
ENROLLMENT						
Inclusion/exclusion criteria		√				
Informed consent		√				
Medical history		√		√	√	
Random allocation			√			
INTERVENTIONS						
TCM Manipulation				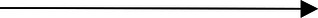		
Schroth Therapy				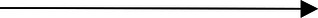		
Home-based Schroth program						√
ASSESSMENTS						
x-Ray		√				√
ATR		√			√	
IR (*Δ*T)		√			√	√
SRS-22r		√			√	√
Adverse events				√		√
Safety evaluation					√	√

The Ethics Committee of Yibin Traditional Chinese Medicine Hospital has given the study approval [Approval No. KY2024 Review (016)]. The study has also been registered at the website of the International Traditional Medicine Clinical Trial Registry on January 6, 2025. The study will adhere to the tenets of the Declaration of Helsinki (Edinburgh 2000 revision).

### 2.2 Participants

#### 2.2.1 Inclusion criteria

Eligible participants who meet the following criteria will be included:(i) Diagnosed with AIS; (ii) Cobb angle between 10° and 45°; (iii) Age 10–18 years, any gender; (iv) Able to provide informed consent (parental/guardian consent where applicable) and comply with follow-up; (v) Able to undergo interventions, spinal radiography, and infrared thermography; (vi) Not currently in a scoliosis bracing program or requiring immediate bracing at the time of enrollment. (vii) No other concurrent scoliosis treatment during study period.

#### 2.2.2 Exclusion criteria

Participants meeting any of the following criteria will be excluded: (i) Severe spinal deformity affecting cardiopulmonary function or requiring immediate surgical correction; (ii) Congenital spinal anomalies or other structural pathology (e.g., spondylolisthesis, tumor, tuberculosis, fracture) incompatible with loading/manipulation; (iii) Severe primary systemic diseases (cardiovascular, pulmonary, renal, hematologic, rheumatic, neuromuscular); (iv) Prior spinal corrective surgery or non-operative treatment within past 3 months; (v) Skin conditions preventing manual manipulation; (vi) significant psychiatric disorders or known poor compliance; (vii) Other conditions precluding participation or protocol adherence.

### 2.3 Interventions

#### 2.3.1 TCM manipulation and Schroth therapy

**Figure 2:**
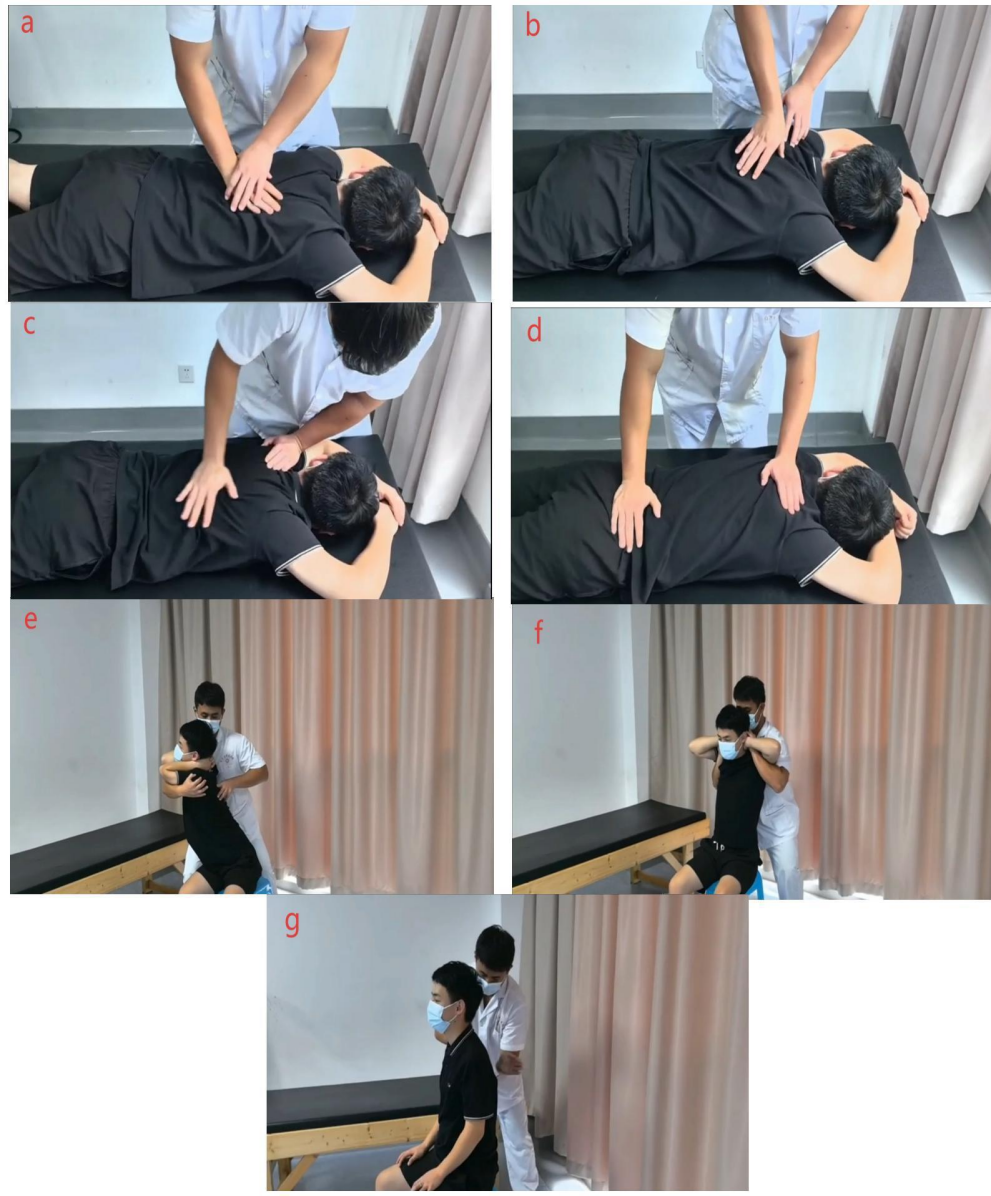
TCM manipulation procedures. **(a)** Gunfa (rolling) and Zhang Roufa (palm kneading) are applied along the Bladder Meridian and the Jiaji region to relax paraspinal soft tissues. **(b)** Yizhichan Tuifa (one-finger Zen pushing) with Zhi Roufa (finger kneading) is performed on selected acupoints to induce a tolerable aching or distending sensation. **(c)** Zhang Ca Fa (palm rubbing) and Niejifa (spinal pinching) are applied along the Du Mai, Jiaji, and Bladder Meridian regions to warm the tissues and stimulate local circulation. **(d)** Rhythmic spinal oscillation is performed with both hands to mobilize spinal segments within the patient's tolerance. **(e)** A seated rotational and extension stretch is guided by the practitioner to promote three-dimensional spinal alignment. **(f)** A bilateral axillary lift with thoracic distraction is used to gently distract and open the thoracic spine. **(g)** The procedure concludes with gentle tapping or kneading to relax the tissues. A detailed step-by-step description of these procedures is provided in Table 2, and a full instructional video has been uploaded as Supplementary Material.

Participants in the intervention group will receive standardized TCM manipulation immediately before each supervised Schroth session during the initial 2-week inpatient phase. Both interventions will be delivered twice daily, five days per week: Schroth sessions will last approximately 2 h, and each TCM manipulation will last approximately 30 min. The TCM protocol is standardized to ensure reproducibility and delivered by licensed practitioners with over five years of clinical experience. Techniques target paraspinal soft tissues, acupoints along the Bladder Meridian, and spinal mobilization, aiming to reduce myofascial tension, enhance segmental mobility, and facilitate three-dimensional postural correction (Table 2, Figure 2).

**Table 2:** TCM manipulation.

Step	Manipulation Technique	Procedure Description
1	*Gunfa* (Rolling) and *Zhang Roufa* (Palm Kneading)	Patient in prone position. Apply rolling and palm kneading to the muscles along the Bladder Meridian of the back and the *Jiaji* region, moving back and forth several times. Palpate for *jingbi* (tender/obstructed) points. Use *Roubo Fa* (kneading and plucking) to relax tense muscles; use rolling and palm kneading on soft or weak musculature. Duration: ∼10 min.
2	*Yizhichan Tuifa* (One-finger Zen Pushing) with *Zhi Roufa* (Finger Kneading)	Based on syndrome differentiation, select acupoints such as *Feishu* (BL13), *Xinshu* (BL15), *Ganshu* (BL18), *Pishu* (BL20), *Weishu* (BL21), *Shenshu* (BL23), *Dachangshu* (BL25). Push along the point using the one-finger Zen technique, pausing at each point for finger kneading 1–2 min, until a tolerable aching/distending sensation occurs. Duration: ∼5 min.
3	*Zhang Ca Fa* (Palm Rubbing) and *Niejifa* (Spinal Pinching)	Apply a small amount of Vaseline or similar medium along the *Du Mai* (Governor Vessel), *Jiaji*, and Bladder Meridian areas. Perform palm rubbing in straight, back-and-forth movements until the skin is slightly red and warm, then pinch along the spine for 3–5 passes.
4	Rhythmic Spinal Oscillation	Place one palm over the cervicothoracic junction or a selected spinal segment for stabilization, and the other palm on the lumbosacral midline. Using the spine as the axis, both palms work together to rhythmically oscillate the spine and pelvis, gradually increasing amplitude within patient tolerance.
5	Seated Rotational and Extension Stretch	Patient seated, arms crossed hugging a pillow, with lower limbs and pelvis stabilized. Practitioner stands behind and slightly to the side, placing one hand on the convex side of the curve for support and the other under the axilla to grasp the opposite shoulder. Guide the patient into slight forward flexion, then rotation and extension toward the same side. Repeat for the opposite side.
6	Bilateral Axillary Lift and Thoracic Distraction	Practitioner passes both arms under the patient's axillae, grasping the upper arms, and gently lifts upward and backward to distract and stretch the thoracic spine, producing mild joint loosening and widening of intervertebral spaces.
7	Finishing Technique	Conclude with gentle tapping or kneading to relax tissues. Total treatment duration: approximately 30–40 min per session.

The intervention group has an additional ∼30 min of therapist contact per session due to the TCM manipulation. Both groups, however, receive the same number of sessions and equivalent follow-up phone support to promote adherence, to partly balance overall attention.

#### 2.3.2 Schroth therapy alone

Participants in the control group will undergo Schroth therapy only. During the initial 2-week inpatient phase, they will receive twice-daily supervised Schroth sessions, five days per week, each lasting approximately 2 h, administered by certified Schroth therapists. This will be followed by a 10-week home-based Schroth program performed three times per week. The intervention includes individualized three-dimensional corrective exercises, sensorimotor training, postural education, and corrective breathing techniques, as summarized in Table 3.

**Table 3:** Schroth therapy.

Step	Exercise Component	Procedure Description
1	Postural Awareness Training	Patient is guided to recognize their scoliosis curve pattern using mirrors and verbal cues. Education is provided on three-dimensional scoliosis correction principles (derotation, deflexion, elongation).
2	Corrective Breathing (Rotational Angular Breathing, RAB)	Patient performs segmental breathing into the concave side of the ribcage while maintaining corrective posture, emphasizing expansion of collapsed areas and active exhalation to promote rib derotation.
3	Pelvic Alignment and Stabilization	Exercises to correct pelvic tilt and rotation are performed in standing, sitting, or supine positions, using isometric activation of gluteal and abdominal muscles to stabilize the pelvis.
4	Spinal Elongation and Axial Stretching	Patient actively lengthens the spine against gravity (in seated or standing positions), with manual or elastic band assistance to increase vertebral spacing and reduce curve compression.
5	Asymmetric Strengthening	Targeted strengthening of trunk muscles on the convex side to support curve correction, using resistance bands or body weight.
6	Symmetric Integration Exercises	Functional activities performed in corrected posture (e.g., squatting, stepping, reaching), reinforcing muscle memory of the corrected alignment.
7	Relaxation and Recovery	Gentle stretching and breathing exercises to relax overactive musculature and prevent fatigue after corrective exercises.

Following discharge, participants will continue with a 10-week home-based Schroth program, performed three times per week under remote monitoring and guidance. To promote adherence, participants will receive weekly follow-up phone calls and are required to record each session in a structured exercise diary, which will be reviewed by the study team. No additional TCM manipulation will be provided during the home phase. Concomitant care such as routine daily activities and non-scoliosis–specific physical activity will be permitted. During the study period, participants will be instructed to avoid any other scoliosis-specific treatments, such as bracing, additional exercise programs, or non-study manual therapies.

#### 2.3.3 Adverse events

Adverse events are defined as any untoward medical occurrence in a participant, and will be categorized by severity (mild/moderate/severe) and relatedness to the intervention. Serious adverse events (SAEs) are defined as events that are life-threatening, require/prolong hospitalization, or result in significant disability or harm.

Adverse events will be systematically assessed at each study visit. Participants will be specifically asked about musculoskeletal discomfort, skin reactions, and neurological symptoms. All adverse events will be recorded, graded, and reported to the ethics committee as per institutional policy. Serious adverse events will trigger immediate review. The trial may be terminated based on the findings of this review. Participants who experience trial-related adverse events will receive free emergency medical care by the investigators.

Additionally, disease progression will be closely monitored as a key safety parameter. All participants will be regularly assessed for clinical signs of worsening, such as increased Angle of Trunk Rotation (ATR) or observable postural changes. If significant clinical deterioration is suspected, a follow-up radiograph will be obtained to confirm progression. Upon confirmation of radiological or significant clinical worsening, the study protocol includes provisions to withdraw the patient from the trial and ensure they receive appropriate standard care.

### 2.4 Randomization and allocation concealment

The allocation sequence will be generated using a permuted block design (random block sizes of 4 and 6) and will be stratified by baseline curve severity (Cobb angle ≤25° vs. >25°) to ensure balance in scoliosis severity between groups. This stratified block randomization will be implemented by an independent statistician. To ensure concealment, the sequence will be embedded into a sequentially numbered, opaque, sealed envelope (SNOSE) system, which will be prepared and safeguarded by an independent research coordinator. After baseline assessment, the coordinator will open the next consecutive envelope to assign the participant to a group. This process will ensure that recruiters and therapists remain unaware of the allocation sequence until after the participant has been assigned to a group.

### 2.5 Blinding

Given the overt nature of the manual therapy intervention, blinding of participants and therapists will not be feasible. To mitigate ascertainment bias, outcome assessors (for radiographic, thermographic, and patient-reported outcomes) and the data analyst will be blinded to group assignment. Allocation will be concealed from these personnel through the use of non-identifiable participant codes until the completion of the statistical analysis. Unblinding of outcome assessors or the data analyst will not be permissible unless required for safety reasons or by the ethics committee, and any such unblinding will follow predefined institutional procedures.

### 2.6 Outcomes

To minimize radiographic exposure, outcome measures will be assessed at different timepoints based on their invasiveness and clinical practicality. All outcomes will be assessed at baseline (week 0) and study completion (week 12). Additionally, to capture the immediate effects of the intensive inpatient intervention, all secondary outcomes will also be assessed immediately following the 2-week intensive intervention period (week 2).

#### 2.6.1 Primary outcome

Change in the Cobb angle (degrees) of the major structural curve (defined as the curve with the largest Cobb angle), will be measured on full-length standing anteroposterior spine radiographs at weeks 0 and 12 only. A fixed tube-to-detector distance of 200 cm, with pelvic leveling blocks used if necessary to compensate for leg length discrepancy. All measurements will be performed by two independent readers blinded to participant allocation and time point. A consensus process with a third adjudicator is predefined for discrepancies greater than 3°. The intra- and inter-rater reliability of the measurements will be formally assessed using intraclass correlation coefficients (ICCs).

#### 2.6.2 Secondary outcomes

Angle of Trunk Rotation (ATR), will be measured by a trunk rotation measurement device (weeks 0, 2, and 12). Axial trunk rotation (ATR) was measured using a Bunnell scoliometer (Hengjian Medical Technology Co., Ltd., Zhengzhou, China). Participants performed the forward-bending Adams test. The trunk rotation angle was systematically measured at the thoracic, thoracolumbar, and lumbar levels, and the values for each segment were recorded separately (14). A minimum of two measurements were taken for each segment per participant, and the average value was calculated and used to improve accuracy. All ATR measurements were performed by the same trained assessor.

Paraspinal surface temperature difference (ΔT) between convex and concave sides will be assessed via infrared thermography at standardized anatomical regions, including the trapezius, latissimus dorsi, and quadratus lumborum muscles (weeks 0, 2, and 12). Imaging will be performed using a short-focus uncooled far-infrared thermographic camera (Baotonghua Medical Equipment Co., Ltd., Chongqing, China). The examination room will be maintained at a constant temperature of 24 ± 2 °C with no drafts. The camera will be actived for 15 min prior to each session. Participants will acclimatize for 15 min while adopting a standardized standing posture (back to camera, head slightly lowered, feet shoulder-width apart, arms slightly abducted). Black body calibration will be performed prior to each participant. The *Δ*T for each muscle will be calculated by subtracting the mean concave-side temperature from the convex-side temperature.

Health-related quality of life, evaluated using the Society-22r(SRS-22r) questionnaire (weeks 0, 2, and 12).

### 2.7 Sample size

The sample size was determined *a priori* using G*Power software (version 3.1.9.7). Based on the primary outcome of change in Cobb angle, an effect size of 0.81 was derived from a previous randomized controlled trial (15). This effect size corresponds to a between-group difference of approximately 4°–5° in Cobb angle improvement. We consider this difference clinically meaningful as it exceeds both the typical measurement error (∼3°) and the established minimal clinically important difference (MCID) of 3.5° for non-operative interventions in AIS (16). The calculation was performed for a two-tailed independent-samples *t*-test, with an alpha (α) error level set at 0.05 and a beta (β) error level set at 0.20 (statistical power of 80%). The result indicated that a total of 50 participants (25 per group) would be required to detect the anticipated effect. To account for a potential attrition rate of approximately 20% throughout the trial period, the target sample size was increased to 60 participants (30 per group) to ensure adequate statistical power for the final analysis.

### 2.8 Data collection and monitoring

Designated outcome assessors, blinded to assignment, will record all measurements on case report forms (paper) and subsequently enter data into a secure electronic database (computerized CRFs). The Academic Committee of Yibin Traditional Chinese Medicine Hospital will function as the independent Data Monitoring Committee (DMC), supervising trial conduct and participant safety. The DMC is independent of the sponsor and the funder. No formal interim efficacy analysis is planned given the sample size. However, the DMC will conduct ongoing safety monitoring. In the event of any safety concerns or unexpected serious adverse events, the DMC is empowered to recommend trial modification or early termination. Data quality audits will be conducted monthly by independent monitors. The statistician performing the outcome analysis will remain blinded to group allocation until the primary analysis is complete. To promote participant retention, reminder phone calls and follow-up messages will be provided. For participants who discontinue or deviate from the intervention protocol, outcome data will continue to be collected at all scheduled time points whenever possible.

### 2.9 Statistical analysis

All analyses will be conducted using SPSS (version 20.0, SPSS Inc., Chicago, IL, USA) with a two-sided significance level of *α* = 0.05. All analyses will follow the intention-to-treat(ITT) principle. Baseline characteristics will be summarized descriptively. The primary outcome, change in Cobb angle from baseline to week 12, will be compared between groups using Analysis of Covariance (ANCOVA), adjusting for the baseline Cobb angle. Results will be presented as adjusted mean differences with 95% confidence intervals. For secondary outcomes measured at multiple timepoints (ATR, ΔT, SRS-22r), linear mixed-effects models (LMMs) will be used to analyze the longitudinal data. The models will include fixed effects for time, group, and their interaction term, with participant as a random effect. The interaction effect will be examined to test if the change over time differs between groups.

Given the multiple secondary outcomes, we will control for multiple comparisons using the False Discovery Rate (FDR) approach (Benjamini-Hochberg procedure) across the secondary endpoints. Secondary outcome *p*-values will be adjusted using FDR at the 5% level to mitigate Type I error inflation. We will treat these analyses as exploratory; any inferences from secondary outcomes will be made with caution.

Missing data will be handled under the missing-at-random assumption using full information maximum likelihood (FIML) in the mixed-effects models. A multiple imputation approach will be performed as a sensitivity analysis to verify the robustness of results.

## 3 Discussion

This protocol describes a randomized controlled trial designed to evaluate the efficacy of integrating Traditional Chinese Medicine (TCM) manipulation with Schroth exercises in adolescents with idiopathic scoliosis (AIS). The rationale stems from two key gaps in the literature: although Schroth therapy has demonstrated moderate efficacy in improving curve magnitude and quality of life, existing systematic reviews highlight the need for more rigorous, high-quality trials due to methodological limitations in prior studies (5, 17–19). Meanwhile, evidence supporting TCM or manual therapy interventions for AIS remains scarce and inconclusive (20, 21). This trial is among the first rigorously designed RCTs investigating the synergistic effects of these conservative modalities.

The study's innovation lies in its integrative approach and inclusion of mechanistic outcomes. Combining scoliosis-specific exercises with manual therapy addresses the hypothesis that preparatory modulation of paraspinal soft tissues optimizes corrective training. Use of infrared thermography as an exploratory biomarker offers potential objective physiological correlates to clinical changes.

The possible mechanisms of benefit are multifactorial. Schroth exercises have been shown to reduce Cobb angle, trunk rotation, and improve muscle endurance and quality of life compared with standard care (15, 22). Manual therapies may enhance outcomes by reducing muscle hypertonicity, improving joint mobility, and modulating autonomic activity. Emerging randomized or controlled studies of combined protocols also report additional benefits when manual therapy is added to physiotherapeutic scoliosis-specific exercises (8, 23). TCM modalities such as acupuncture and massage have further been reported to reduce curvature and pain severity in AIS (11, 24). These clinical observations are consistent with physiological evidence that massage-type interventions can increase local perfusion, reduce soft-tissue stiffness, and influence autonomic balance, thereby creating a more favorable milieu for corrective neuromuscular retraining (25). When combined with the neuromuscular re-education provided by Schroth methods, the synergistic potential is therefore plausible.

Clinically, this study may broaden non-surgical options for AIS. While bracing and exercise remain mainstays, their limitations in compliance and acceptability are well recognized (4). An adjunct such as TCM manipulation—often well-tolerated and culturally acceptable—could improve adherence and outcomes when integrated into rehabilitation. If effective, findings may support broader consideration of manual therapy within scoliosis management pathways, particularly in integrative medicine contexts.

Several limitations should be acknowledged. One limitation is the inability to blind participants and the greater contact time in the intervention arm. This could introduce an attention or placebo effect biasing patient-reported outcomes. We attempted to mitigate this by providing equal frequency of sessions and follow-up in both groups, but a residual difference in therapeutic contact remains and must be considered when interpreting results. Second, the 12-week follow-up may not reflect long-term durability of improvements or impact on surgical risk. Third, being a single-center trial, operator-dependent variability and limited generalizability are concerns. Finally, we recognize that standard care for certain AIS patients (curve >25°) includes bracing. Our study intentionally excludes bracing to assess the interventions in isolation, which may limit generalizability. This design was chosen with careful ethical consideration: the trial is short-term, and participants are closely monitored so that if any significant progression occurs, they can transition to bracing or other needed care promptly.

Future studies should aim for multi-center trials with longer follow-up through skeletal maturity. Larger samples would enable subgroup analyses (e.g., by curve type or severity), while mechanistic investigations (e.g., surface EMG, advanced imaging, biomarkers) could elucidate how manual therapy augments exercise effects. Economic evaluations are also recommended to assess scalability and resource implications (15).

## 4 Conclusion

In conclusion, this trial aims to provide high-quality evidence on the efficacy and mechanisms of an integrative, non-invasive strategy combining Schroth exercises with standardized TCM manipulation for AIS. Its findings may inform clinical practice and future guideline development in the management of idiopathic scoliosis.

## Research status

The recruitment of participants for this study was initiated on June 1, 2025. As of the present date, recruitment remains active and is expected to conclude by December 31, 2025. No findings from this study have been disseminated or submitted for publication in any form.

## Data sharing plan

De-identified individual participant data (IPD), along with the data dictionary and statistical analysis code, will be made available beginning 6 months after the publication of the primary results and for a period of 3 years. Data access will be granted to qualified researchers who provide a methodologically sound research proposal that has received approval from an independent ethics committee. Proposals should be submitted to the Corresponding Author for review by the trial steering committee. A signed data access agreement will be required before data are shared through the secure institutional repository maintained by Yibin Traditional Chinese Medicine Hospital.
